# PCSK9 vaccines: a promising new strategy for the treatment of hypercholesterolemia?

**DOI:** 10.1016/j.jlr.2024.100524

**Published:** 2024-02-17

**Authors:** Bryce Chackerian, Alan T. Remaley

**Affiliations:** 1 Department of Molecular Genetics and Microbiology, University of New Mexico School of Medicine, Albuquerque, NM, USA; 2 Lipoprotein Metabolism Laboratory, Translational Vascular Medicine Branch, National Heart, Lung, and Blood Institute, National Institutes of Health, Bethesda, MD, USA

Management of elevated LDL-C, a causal risk factor in the development of atherosclerotic cardiovascular disease (ASCVD), has been revolutionized by using inhibitors of proprotein convertase subtilisin/kexin type 9 (PCSK9). When used in conjunction with statins, PCSK9 inhibitors can markedly lower LDL-C to levels that have not been previously attained with other lipid-lowering medications.

Circulating LDL-C is normally cleared from plasma when it taken up by hepatocytes that express the LDL receptor (LDL-R). PCSK9, a secretory protein largely produced by the liver, negatively regulates LDL-C homeostasis by mediating the internalization and degradation of LDL-R. Naturally occurring mutations that increase PCSK9 activity are associated with higher levels of circulating LDL-C, whereas mutations that downregulate PCSK9 lead to low serum LDL-C levels and a lower risk of ASCVD. Currently, two classes of PCSK9 inhibitors have been approved by the US Food and Drug Administration. These include two anti-PCSK9 monoclonal antibodies (mAbs), evolocumab and alirocumab, and an siRNA-based therapeutic, inclisiran. In hypercholesterolemic individuals these drugs can dramatically lower LDL-C levels, often by as much as 60% or more (1, 2, 3). The widespread adoption of these PCSK9 inhibitors, however, has been relatively slow, largely due to their high cost. Consequently, PCSK9 inhibitors are reserved for high-risk patients and for secondary prevention for those patients who fail to achieve target LDL-C levels, using conventional statin-based therapies (4). Another limitation is that anti-PCSK9 mAbs require regular subcutaneous injections every 2–4 weeks, which complicates their utilization and reduces patient adherence. These practical issues have prompted efforts to develop more accessible and/or longer lasting alternatives for inhibiting PCSK9, such as an oral drug inhibitor (5), a gene editing approach (6), and vaccines targeting PCSK9 (7, 8, 9).

In this issue of the *Journal of Lipid Research*, Vroom and colleagues report the results of an efficacy study of a promising PCSK9 vaccine candidate in nonhuman primates (8). VXX-401 is a peptide-based vaccine that elicits targeted antibody responses against an epitope in the catalytic domain of PCSK9. Although vaccines are an established and effective intervention for infectious diseases, the development of a vaccine targeting PCSK9 presents unique challenges due to the inherent difficulties in eliciting immune responses against self-antigens. VXX-401 overcomes immune tolerance mechanisms by physically linking a PCSK9 peptide to a strong T helper epitope derived from a foreign antigen and combining this protein vaccine with a potent AdjuPhos/CpG1 adjuvant cocktail. VXX-401 elicited high-titer anti-PCSK9 antibodies in macaques that resulted in a ∼30–40% reduction in plasma LDL-C relative to a control group of unvaccinated animals. Importantly, immunization with VXX-401 did not affect HDL-C levels and was not associated with any adverse events, such as chronic inflammation or uncontrolled autoimmunity against PCSK9. The lack of adverse effects upon vaccination in this study suggests that inducing antibodies against PCSK9 may be a safe approach.

VXX-401 is one of several PCSK9 vaccines that are currently under development. AT04A and AT06A are peptide-based vaccines developed by AFFiRiS AG that have already undergone phase I clinical trials (9). These vaccines also link a PCSK9-like peptide to a foreign carrier protein, but the PCSK9 sequences have been modified from the native human PCSK9 sequence by one (AT04A) or two (AT06A) targeted amino acid substitutions. The rationale underlying this strategy is that a nonself peptide may be better able to overcome self-tolerance but could still elicit antibodies that cross-react with native PCSK9. In the phase I clinical trials, these vaccines were administered with Alhydrogel adjuvant and were safe and well tolerated. However, only AT04A decreased LDL-C levels, and while its LDL-C lowering effects were only modest (11%–13% reductions), a sustained reduction of LDL-C at this level would likely be beneficial in reducing ASCVD events (10). We have also developed a PCSK9 vaccine takes advantage of the observation that displaying self-antigens on the surface of a highly repetitive, multivalent structure can efficiently overcome B cell tolerance. Using a bacteriophage virus-like particle–based vaccine platform, we generated a bivalent vaccine that targets two epitopes within PCSK9, 153–163 aa (which was targeted by both VXX-401 and AT04A) but also a second epitope on PCSK9 (207–223 aa) that also interacts with the LDL-R. In nonhuman primates, the bivalent vaccine (administered with Alhydrogel) was well-tolerated, elicited strong anti-PCSK9 antibody responses, and reduced LDL-C by ∼30% (7).

Vaccination, unlike current mAb and siRNA therapies, is typically an affordable intervention, which potentially allows a broader population to access PCSK9 inhibitors. Vaccines can also be readily distributed globally, allowing an avenue to address ASCVD in regions of the globe that may not only lack access to advanced therapies but even statins, which are now off patent and relatively inexpensive. Vaccines also have the potential to stimulate prolonged immune responses, potentially obviating the need for frequent administrations, which could lead to improve patient compliance, a major issue for any type of therapy. Interestingly, the longevity of antibody responses elicited by the three leading PCSK9 vaccine candidates varied. VXX-401 elicited antibodies with a relatively short half-life, just over 2 weeks, and the LDL-C lowering effects of vaccination with VXX-401 waned as anti-PCSK9 IgG levels declined (8). In contrast, antibodies elicited by vaccination with AT04A (in humans) had a half-life of 12 weeks (9) and anti-PCSK9 antibodies elicited by the virus-like particle–based PCSK9 vaccine had a half-life of 20 weeks (in mice) (7). It is unclear why these differences in antibody half-life exist but it may stem from the vaccine technology and/or the specific adjuvants employed. Nevertheless, it is likely that even PCSK9 vaccines will require regular boosting to sustain LDL-C lowering effects. Indeed, a booster immunization with VXX-401 was effective at restoring anti-PCSK9 antibody titers to peak levels and reducing LDL-C (8). Thus, successful implementation of a PCSK9 vaccine will ultimately require a comprehensive understanding on the precise antibody levels required to achieve therapeutic effects and possibly new approaches for addressing the variability of immune responses amongst individuals. In summary, much more research and developmental work still needs to be done for PCSK9 vaccines, but at this time they appear to have great potential to adding to our lipid-lowering armamentarium for the prevention of cardiovascular disease.

## Conflict of interest

The authors are inventors of VLP-based vaccines targeting PCSK9.
